# Update on traumatic brain injury in the ICU

**DOI:** 10.1097/ACO.0000000000001468

**Published:** 2025-02-12

**Authors:** Rosalinde E.R. Slot, Raimund Helbok, Mathieu van der Jagt

**Affiliations:** a Department of Intensive Care Adults, Erasmus MC, University Medical Center, Rotterdam, The Netherlands; b Department of Neurology; c Clinical Research Institute of Neuroscience, Johannes Kepler University Linz, Kepler University Hospital, Linz, Austria

**Keywords:** brain tissue oxygen tension management, intensive care, intracranial pressure, personalized medicine, severe traumatic brain injury

## Abstract

**Purpose of review:**

This review aims to summarize recent developments for the management of severe traumatic brain injury (TBI) in the ICU. Recent advancements in TBI ICU management emphasize a progression toward more multimodal approaches and mitigating secondary brain injury by increased focus on careful systemic management.

**Recent findings:**

Invasive monitoring techniques such as continuous intracranial pressure (ICP) and brain tissue oxygen pressure (PbtO_2_) monitoring are considered standard of care or may become crucial, respectively, for managing severe TBI. Technological advances in noninvasive techniques (e.g. quantitative pupillometry) are likely to advance our diagnostic and prognostic ability. Blood biomarkers, including glial fibrillary acidic protein, neurofilament light chain, and ubiquitin carboxy-terminal hydrolase L1, provide minimally invasive ways to better assess injury severity and predict outcomes. These advancements support personalized care, which will likely influence clinical management strategies in the future.

**Summary:**

ICP monitoring remains a key component of severe TBI management in ICU. Emerging evidence is slowly changing and improving intensive care and patient outcomes and include both brain-targeted therapies and careful systemic intensive care management.

**KEY POINTS**

- **Neuromonitoring**: invasive intracranial pressure (ICP) monitoring plays an important role in traumatic brain injury (TBI) management, with dual monitoring (ICP + brain tissue oxygen pressure) offering additional information to improve patient management. Two large international trials are ongoing that should provide definitive answers in the years to come. Metrics such as Pressure Reactivity Index are emerging as important tools to assess cerebrovascular reactivity, potentially allowing for individualized management of cerebral perfusion pressure.
- **Blood biomarkers**: biomarkers such as GFAP, NFL, UCH-L1, and S100B are valuable in assessing injury severity and predicting outcomes in TBI. Studies suggest that serial measurements of these biomarkers could help predict long-term outcomes and guide treatment decisions, especially when clinical observations are unreliable. Studies focused on biomarker-based management algorithms should be done to prove clinical value.
- **ICU management**: effective management of TBI in the ICU includes a liberal transfusion strategy, while still avoiding both hypovolemia and fluid overloading. The optimal management strategies are described by the Seattle (Seattle International Severe Traumatic Brain Injury Consensus Conference) consensus guidelines and the recent American College of Surgeons Trauma Program Best Practice Guidelines.
- **Future perspectives**: machine learning models hold promise for predicting ICP crises and other adverse events in TBI. However, these models require further clinical validation to ensure reliability and integration into clinical practice.

## INTRODUCTION

Traumatic brain injury (TBI) remains a leading cause of morbidity and mortality globally, with millions affected annually and substantially impacting healthcare systems [1]. Management of TBI in the ICU is a complex and multifaceted process that requires continuous and precise monitoring of both intracranial and systematic pathophysiology. In recent years, advancements in neuromonitoring, prognostication, and biomarker research have enhanced our understanding and management of TBI. In this review, we summarize recent developments pertaining to noninvasive and invasive ICP monitoring techniques, blood biomarkers, and general ICU management strategies, including fluid and transfusion management.

## MONITORING TRAUMATIC BRAIN INJURY IN THE ICU

### Severity grading and the value of repeated neurological assessments

Repeated clinical assessment is the foundation of neuromonitoring in TBI patients. The value of the Glasgow Coma Scale (GCS) is limited in sedated patients as it lacks precision for prognostication, except for the motor (M) subscore [2]. The GCS is still used in the current classification of TBI (mild: GCS 13–15, moderate: GCS 9–12, or severe TBI: GCS ≤8), although recent studies suggest that integration of systemic metabolic parameters (e.g. blood glucose and renal function) may detect specific TBI phenotypes and aid in better prognostication models [3^▪^]. In addition, the International Initiative for Traumatic Brain Injury working group proposed to include clinical (C), imaging (I), and blood (B) derived biomarkers (C-B-I) to characterize injury severity as well as, that is, premorbid health and socioeconomic factors (CBI-M; M for modifiers [4^▪▪^]. Integrating quantitative pupillometry (QP) in the acute phase has added diagnostic and prognostic information in patients with TBI, and should be documented throughout the acute phase [5]. QP is more accurate than standard clinical evaluation of pupillary size and reactivity and may provide early warning information before clinical deterioration, for example, ICP increase [6^▪^]. This can be important especially when opioids and sedative drugs result in small pupils and hamper clinical examination.

### Intracranial pressure monitoring

Currently, no widely used monitoring tool can reliably supplement invasive ICP monitoring. Noninvasive tools including optic nerve sheath measurement and transcranial Doppler have been studied showing consistent associations with ICP, which, however, are insufficiently accurate to replace invasive ICP monitors [7]. However, these monitoring techniques may provide supportive information in individual patients (e.g. ruling out increased ICP), especially in low- and middle-income countries where invasive ICP monitoring is not always available [8]. ICP monitoring is recommended to improve outcomes in comatose patients (GCS ≤ 8) with structural brain damage on initial computed tomography (CT), as suggested in the latest guidelines from the American College of Trauma Surgeons, where previous guidelines were less unambiguous [9^▪▪^]. However, there is still significant variability in the use of ICP monitoring among trauma centers in Europe and worldwide. Placement of an external ventricular drain is the gold standard for ICP assessment, still, most centers use an intraparenchymal transducer targeting ICP values below 20–25 mmHg. ICP monitoring has been associated with a more intensive therapeutic approach, but with lower 6-month mortality, especially in more severe cases in a large observational study (SYNAPSE) [5]. ICP management strategies should follow the Seattle International Severe Traumatic Brain Injury Consensus Conference (SIBICC) protocol which provides stepwise management recommendations, which may be adapted to local environment [10^▪▪^].

Continuous ICP monitoring can be supplemented by brain tissue oxygen pressure (PbtO_2_) monitoring, which is a surrogate marker of cerebral blood flow (CBF). The added value lies in the detection of brain tissue hypoxia, even in the absence of high ICP, which has been shown to be associated with worse outcome [11,12^▪^,^13^]. Consensus recommendations for clinical use were recently published and – when implemented - can be adapted to local circumstances [9^▪▪^,^14^^▪▪^]. The added benefit of oxygen monitoring on patient outcome remains to be determined after several single-center studies and a phase 2 clinical trial proved safety and suggested outcome benefit [13,15^▪^]. Recently, the OXY-TC trial was published, studying whether dual monitoring (ICP and PbtO_2_, with standardized clinical protocol to improve brain tissue hypoxia), versus ICP monitoring alone improved neurological outcomes after 6 months [16^▪▪^]. Dual monitoring (ICP and PbtO_2_) followed by protocolized treatment of brain tissue hypoxia did not improve neurological outcome. However, post hoc analyses indicated a potential benefit for dual monitoring in patients with high initial ICP (≥20 mmHg). The dual-monitoring group had a slightly higher incidence of adverse events, including catheter dysfunction and intracerebral hematoma, which may point to intrinsic complications associated with the dual monitoring, insufficient experience with it, or both. Ongoing large trials investigating PbtO_2_, next to ICP monitoring, should provide more robust evidence on the added value of this modality regarding clinical outcome (BOOST-3 and BONANZA). Combining information from ICP monitoring and PbtO_2_ measurement may not only aid in guiding therapeutic interventions to reduce brain hypoxia but also inform timing and need of decompressive craniectomy (DC). New questions will likely arise over time, for example, whether high ICP (20–25, or even up to 30 mmHg) may be acceptable when tissue hypoxia is absent, which may challenge current paradigms.

### Blood biomarkers in traumatic brain injury

Blood biomarkers in moderate-to-severe TBI may provide objective and additional information on injury severity and prognosis when confounding factors (e.g. sedation or systemic insults on admission) are present. Several markers of injury are identified, for example, markers of glial injury, such as glial fibrillary acidic protein (GFAP), S100 calcium-binding protein B [S100B], and neurofilament light chain (NFL), and markers of neuronal injury ubiquitin carboxy-terminal hydrolase L1 (UCH-L1) and neuron-specific enolase (NSE). Rapid measurement of these biomarkers was recently authorized by the Food and Drug Administration for routine clinical use in the USA, and in Scandinavian countries, S100 B has already been incorporated in clinical guidelines for mild TBI to rule out the need for CT imaging since 2013. GFAP and UCH-L1 are other sensitive biomarkers that may be used to rule the need for a brain CT scan [9^▪▪^]. In the CENTER-TBI cohort, higher concentrations of GFAP, NFL, and UCH-L1 within the first 24 h after TBI were associated with higher levels of disability and mortality in all injury severities of TBI [17^▪^]. In a large review of several types of biomarkers, GFAP, UCH-L1, and S100B were associated with TBI severity and strong predictors of functional outcome in TBI at 6 months. NFL, NSE, and tau were also of interest, but associations with outcome were less strong [18]. In another study from the CENTER-TBI cohort, disease trajectories of 1728 patients with severe TBI in the ICU were analyzed. UCH-L1, GFAP, S100B, NSE, NFL, and tau were reliable predictors of outcome, with higher levels associated with worse outcome [3^▪▪^]. Authors suggested that serial measurements of these biomarkers during ICU stay may enhance predictive accuracy and enable closer monitoring of disease progression in TBI patients. For clinical practice, blood biomarkers may soon become easily accessible and more widely used markers, that not only aid in predicting long-term outcome and decision-making in mild TBI but may also guide initial severity grading informing triage on admission: for example, which patients should have a wake-up call in the emergency department versus which patients should have ICP monitoring at the ICU. However, more studies are warranted before widespread clinical implementation.

## ICU MANAGEMENT OF TRAUMATIC BRAIN INJURY

### Transfusion strategy in traumatic brain injury

The recently published results from the TRAIN study and HEMOTION trial indicated that patients with acute brain injury and anemia randomized to a liberal transfusion strategy were less likely to have an unfavorable neurological outcome [defined as lower Glasgow Outcome Scale Extended (GOS-E)]. The TRAIN study [(Hb <9 vs. <7 g/dl) found an adjusted relative risk of 0.86 (95% confidence interval {CI}, 0.79–0.94); *P* = 0.002] for the restrictive strategy [19^▪▪^]. The HEMOTION trial found an adjusted absolute difference (liberal strategy (≤10 g/dl) vs. restrictive (≤7 g/dl)) of 5.4% (95% CI: −2.9 to 13.7), which did not reach statistical significance, but results were in the same direction as TRAIN [20^▪▪^]. These results are in contrast with most studies on restrictive transfusion strategies in non-TBI ICU patients, but similar to recent results from the international CENTER-TBI study, which indicated a significantly increased risk of unfavorable neurological outcomes and increased mortality in patient with low Hb levels (Hb ≤7.5 g/dl; OR, 3.21; 95% CI, 1.59–6.49) [21^▪▪^].

### Fluid management

The ESICM consensus guidelines on fluid management in acute brain injury were informative for clinical management also in TBI but stem from 2018. An international survey of ICU physicians evaluating fluid management in TBI showed that clinical practice is highly variable between centers and countries [22]. However, previous research showed that aiming for a mean neutral fluid balance (indicating normovolemia) might contribute to improved outcomes in critically ill TBI patients [23]. The practical implications of individualized fluid therapy and maintaining a normovolemic state based on fluid balances and, if needed, additional hemodynamic monitoring were recently reviewed [24^▪^]. Importantly, what constitutes a ‘mean neutral fluid balance’ in otherwise hemodynamically stable TBI patients, was further clarified in relation to previous multicenter studies, as referenced above, since there is much debate on clinical interpretation and implementation of fluid balance-based fluid and hemodynamic management [25].

### Cerebral perfusion pressure and mean arterial pressure

ICP monitoring allows the calculation of cerebral perfusion pressure [CPP = mean arterial pressure (MAP) – ICP], a determinant of cerebral perfusion and blood flow. Assessment of cerebral autoregulation (CA) is recommended in recent guidelines [11] and may help to establish individualized CPP goals. Identifying the CPP range where autoregulation is most effective, may vary among patients and change dynamically over time. The pressure reactivity index (PRx, moving correlation over time between ICP and MAP) [26] has enabled the identification of personalized or population-derived optimal CPP (CPPopt), which might allow individualized blood pressure targets [14^▪▪^]. CPPopt may be higher than the conventional range in some cases, such as in patients with decreased autoregulation and significant brain swelling. Impaired autoregulation is observed in patients with and without elevated ICP and may be integrated into local protocols of specialized centers. However, there is still a lack of high-level evidence in favor of CA-based treatment protocols regarding the effect on clinical outcomes. CPP management in TBI based on CPPopt targets has been shown feasible and safe (COGITATE trial), and this personalized approach is of great interest to further evaluate [27]. Without such advanced monitoring, the most recent guidelines advise to target systolic blood pressure ≥100 mmHg and CPP between 60 and 70 mmHg [9^▪▪^].

### Hyperosmolar therapy

Mannitol and hypertonic saline (HTS) are both used to manage increased ICP, either alone or combined. A recent study from the CENTER-TBI cohort analyzed the impact of preferences for either mannitol, HTS, or both concomitantly in managing ICP in TBI patients. Treatment choices varied mainly by center preference rather than being guided by individual patient characteristics. Further, 57% of patients received HTS as their primary hyperosmolar agent (HOA), 30% received mannitol, and 13% received both agents. There were no significant differences in ICU mortality or 6-month outcomes (GOS-E) between patients treated with mannitol, HTS, or a combination of both agents. Therefore, while both agents can effectively manage increased ICP, using either or both, as is the currently existing variability across trauma centers, appears an acceptable practice variability [28^▪^,^29^]. A large review found no superiority of HTS vs other ICP lowering agents on outcome (GOS), but mentioned an increased risk of severe hypernatremia [30], while another study showed slight benefit of HTS on functional outcome in patients with medium ICP [31]. The prevalence of acute kidney injury (AKI) in patients receiving mannitol and/or HTS may or may not differ: one study showed that after adjusting for factors such as severity of TBI, the hazard ratio for developing AKI was 2.13 for mannitol versus 1.5 for HTS, suggesting a lower AKI risk for treatment with HTS in TBI patients; whereas another study showed no significant difference of AKI prevalence in TBI patients receiving HTS vs no hyperosmolar therapy [29,31]. Further research is ongoing (e.g. the sugar or salt trial [32]).

### Mechanical ventilation

Mechanical ventilation should be aimed at normal PaCO_2_ in TBI, since fluctuations can lead to changes in CBF and intracranial pressure (ICP). Hypocapnia (low CO_2_) causes cerebral vasoconstriction and reduced CBF, whereas hypercapnia (high CO_2_) can increase ICP due to vasodilation and increased CBF [33].

A recent study evaluated the relationship between PaCO_2_ levels and outcomes in acute brain injury patients on mechanical ventilation. Authors observed that PaCO_2_ had a U-shaped association with inhospital mortality, with both severe hypocapnia (<32 mmHg) and hypercapnia (>45 mmHg) linked to increased mortality. Mild hypocapnia (32–35 mmHg) was generally tolerated in TBI patients [34^▪^]. It is common practice to manage lung-protective ventilation (LPV) with low tidal volume (4–6 ml/kg) and varying positive end-expiratory pressure in ICU patients, but the effect of LPV in patients with TBI without ARDS remains unknown. A recent study evaluated LPV in TBI and observed that most patients tolerated LPV without significant increases in ICP, but 22% of patients experienced ICP spikes above 22 mmHg, leading to intervention interruption. LPV did not significantly impact CA, measured with PRx [35]. For clinical practice, it remains important to strive for normocapnia, and LPV wherever possible.

### Temperature management

Fever control should be prioritized, irrespective of ICP status, especially for patients at risk of seizures or cerebral herniation. Fever, whether neurogenic or infectious, is associated with unfavorable outcomes and must be promptly managed to mitigate secondary injury. Temperature control (36.0–37.5 °C) is considered an essential aspect of high-quality TBI care. Continuous core temperature monitoring and fever management are recommended as key measures to prevent secondary brain injury [36^▪^].

### Sedation

Sedation is often used in severe TBI in the ICU for the patients to tolerate mechanical ventilation and to reduce cerebral metabolism and optimize VO2/DO2 balance. Sedation practices vary among centers, with large center and country variation in therapy intensity and type of sedative, including propofol, and midazolam, amongst others [37]. Dexmedetomidine is a selective α2-agonist that decreases sympathetic activity by reducing sympathetic outflow from the nucleus coeruleus. Originating from the idea that early sedation with dexmedetomidine may reduce autonomic dysfunction and inflammation during the acute recovery phase in TBI, the use of dexmedetomidine was investigated in 77 patients with moderate and severe TBI [38]. Early dexmedetomidine exposure was not significantly associated with better 6-month functional outcome (GOS-E) across the entire cohort, but for those patients with ICP monitoring, early dexmedetomidine was associated with higher 6-month GOS-E scores, and shorter hospital stay, suggesting potential benefits in this subgroup. Another recent trial evaluated the use of pentobarbital coma in severe TBI. Pentobarbital coma seems a last resort for high ICP, and is, although used for decades, not well studied. The effects of pentobarbital coma on ICP and its association with functional outcome was analyzed. Results showed that patients who timely responded to pentobarbital coma with lower ICP had better functional outcome. Patients without ICP lowering in response to pentobarbital had worse outcome [39]. In conjunction with the rescue ICP trial (showing that, in medically refractory increased ICP, DC appears superior to a strategy with barbiturates as rescue therapy instead of DC), this may indicate that barbiturates may still have a place in the armamentarium of ICP control but that immediate response of ICP to barbiturates is an important factor [9^▪▪^].

## CONCLUSIONS AND FUTURE PERSPECTIVES

We aimed to provide insight into some recent advances in TBI at the ICU that may inform the practicing physician. Emerging evidence and research indicate that improvements in clinical management and outcome are possible, which helps to tackle therapeutic pessimism in the field of severe TBI. Progression in this field is overall slow but may accelerate in the coming years due to several factors. Large trials are ongoing regarding the effectiveness of dual (ICP/PBtO_2_) monitoring, and these studies will likely change ICP management significantly or deliver insights helping to change practice after many years of relative indolence regarding improved monitoring and therapy for severe TBI. Blood biomarkers show great promise for predicting outcomes and guiding treatment decisions and warrant further validation studies and clinical implementation. ICU management strategies, such as liberal transfusion, normovolemia maintenance, use of hypertonic solutions, and temperature management aim to improve patient outcomes, and clinical evidence underpinning these treatments is progressing (see also Table 1 for an overview of suggestions). Finally, with the increasing collaborations between clinicians and technical experts, new avenues emerge that may impact the field. Machine learning models, for example, to predict ICP crises and enhance decision-making in TBI management, are being explored and are expected to change practice in years to come [40^▪^]; dashboarding of multimodality monitoring signals may be enhanced by AI algorithms, finally facilitating the step towards more widespread implementation; and new trial designs, enabling more flexible patients inclusion and easier workflow of trials (e.g. adaptive platform trials) are expected to improve the chance that clinical trials can change practice and improve outcomes [41]. In the meantime, the implementation of consensus guidelines deserves scrutiny and careful intensive care treatment mostly aimed at ‘normality’ of clinical parameters and avoiding iatrogenic harm (e.g. fluid overload) are of utmost importance to optimize clinical outcomes after severe TBI.

**Table 1.** Practical recommendations for ICU management of traumatic brain injury (original)

Theme	What can you change tomorrow in your practice?	Future perspective
Noninvasive neuromonitoring	Consider using pupillometry as a supplementary neuromonitoring tool	Pupillometry might add to prognostication and the assessment of secondary brain injury
Invasive neuromonitoring	Use published protocols (e.g. SIBICC) as a practical basis for local protocols (available for both ICP and ICP/PbtO_2_ combined monitoring)	Future standard of care may become dual-monitoring (ICP + PbtO_2_) pending ongoing trials
Blood biomarkers	Blood biomarkers for TBI are used in some countries as an aid for decision-making, which patients should receive additional CT scans. They appear to be informative for severity grading on top of clinical and imaging assessment	Incorporation of blood biomarkers (GFAP, S100B, UCH-L1) for enhanced severity grading and prognosis in moderate-to-severe TBI patient might help in triaging patients based on severity and guide initial management
Transfusion strategy	Implement a liberal transfusion strategy, avoid Hb <9 g/dl in TBI admitted to ICU	More personalized transfusion strategies might be facilitated by results of ongoing trials on PbtO_2_ or cerebral autoregulation
Fluid management	Maintain a mean neutral fluid balance in TBI patients	The role of more advanced hemodynamic monitoring, apart from fluid balance assessments, may become more clear pending further studies, also in hemodynamically stable patients
Temperature control	Prioritize fever control to mitigate secondary brain injury, maintaining temperatures between 36.0 and 37.5 °C.	

ICP, intracranial pressure; PbtO_2_, brain tissue oxygen tension; SIBICC, Seattle International Severe Traumatic Brain Injury Consensus Conference; TBI, traumatic brain injury.

### Acknowledgements

*R.E.R.S., R.H., and M.J. contributed to the writing of this article.*

### Financial support and sponsorship

*R.E.R.S.: none. M.J. reports having received honoraria from Integra LifeSciences and funding from the Dutch Brain Foundation. He is a member of the Working Group of Observational and Comparative Effectiveness Studies for the International Initiative of Traumatic Brain Injury (InTBIR) studies. R.H. received fees for lectures from BD, Zoll, Integra, and Neuroptics and reports funding from Austrian Science Fund (FWF) and Fresenius Kabi, all outside the submitted work.*

### Conflicts of interest

*There are no conflicts of interest.*

## REFERENCES

Papers of particular interest, published within the annual period of review, have been highlighted as:

▪ of special interest
▪▪ of outstanding interest
